# Physicochemical, enzymatic and molecular characterisation of the storage protein of aerial tuber, *Dioscorea bulbifera* Linn.

**DOI:** 10.1186/s43141-020-00040-y

**Published:** 2020-07-13

**Authors:** Olukemi Adetutu Osukoya, Adenike Kuku

**Affiliations:** 1 Department of Chemical Sciences, Afe Babalola University, Ado Ekiti, Nigeria; 2 Department of Biochemistry and Molecular Biology, Obafemi Awolowo University, Ile-Ife, Nigeria

**Keywords:** Aerial potato, Aerial yam, Bulb, Dioscorin, Storage protein, Protein purification

## Abstract

**Background:**

The storage protein of the aerial tuber of *Dioscorea bulbifera* was purified and its physicochemical, enzymatic and molecular properties determined with a view to comparing its functionality and genetic relatedness with other storage proteins.

**Results:**

The purified protein had molecular weight of 21 kDa. The protein showed carbonic anhydrase, trypsin inhibitory, dehydroascorbate reductase and monodehydroascorbate reductase activities. Amplifications with polymerase chain reactions resulted in the detection of two genes encoding the storage protein. The deduced amino acid sequence of the shorter and larger genes had homologies with the storage proteins of members of the *Dioscorea* family.

**Conclusion:**

The study concluded that the storage protein of the aerial tuber of *D. bulbifera* had similar properties with those of other *Dioscorea* species and may be suitable for development as functional food.

## Introduction

Yam, a dioecious plant belonging to the *Dioscorea* genus, is an important staple crop in many areas of the tropics and sub-tropics [1]. There are 8 genera and 880 species of yam plant. They produce edible tubers, bulbils, corms or rhizomes [2] that are basically made up of carbohydrates and are important sources of proteins and micronutrients [3]. Yam tubers are widely utilized as food due to their compositions [4]. Yam tubers also contain functional components such as mucin, dioscin, diosgenin, allantoin, choline and polyphenol oxidases [5] and, in addition, minerals and vitamins such as calcium, zinc, phosphorus, copper, iron, sodium, potassium, β-carotene, thiamine, riboflavin and niacin [6]. About 80% of the proteins in yam are storage proteins [7], which are usually affected by factors such as cultural practices, climate, soil fertility, maturity at harvest and length of storage time [8].

Plants accumulate storage substances such as starch, lipids and proteins in certain phases of development. The major role of storage proteins is to act as stores of nitrogen, sulphur and carbon, which are accumulated in both vegetative and reproductive tissues. Thus, they serve as a reservoir for later stages of plant development [9, 10]. Storage proteins provide nutrients to support the growth of new plants as seedlings (from seeds) or shoots (from tubers). They are localized in specific organs, cell types and subcellular compartments in discrete deposits (protein bodies) where they facilitate high-level accumulation without any adverse effects on other cellular functions. They also allow plants to survive periods of adverse conditions between growing season [11]. Plant storage proteins are grouped into two classes: seed storage proteins that accumulate to high levels in seeds during the late stages of seed development, and vegetative storage proteins, which accumulate in vegetative tissues such as leaves, stems and tubers [12].

Dioscorins, yam storage proteins, isolated from different *Dioscorea* species have been shown to have various biological activities, which include enzymatic (α-carbonic anhydrase, trypsin inhibitory), antioxidant, antihypertensive and immunomodulatory activities. They are thus worth developing as healthy or functional foods. *Dioscorea bulbifera* belongs to the family *Dioscorea*ceae assigned to the order Dioscorales. It is commonly known as air potato, potato yam, air yam, or bulbil-bearing yam. It is native to Africa and Asia but widely grown and consumed in the tropics [13], the Caribbean Islands, South East Asia, South Pacific and West Indies. The uncultivated form is bitter, not edible and may be poisonous. Air potato plants produce “aerial tubers” that are attached closely to the axil. These aerial tubers (bulbils) are usually round or spherical with mostly smooth surfaces. The aerial tuber (from where the name ‘air potato’ is derived) serves as the main storage organ of *D. bulbifera* [13, 14]. The aerial tubers of *D. bulbifera* are commonly consumed especially in South Eastern Nigeria and serve as a good source of calories and minerals [15]. The plant has many health benefits and is used in folk medicine as analgesic, aphrodisiac, diuretic and rejuvenative tonic. It is also used as a folk remedy to treat conjunctivitis, diarrhea and dysentery [16, 17]. Despite all its medicinal and agricultural uses, *D. bulbifera* is widely characterized as an organism that outcompetes and smothers native vegetation and is usually considered as weed by farmers. It is thus paramount to investigate the properties of some of the bioactive molecules such as the major storage protein. The study aimed at isolating and purifying the storage protein from the aerial tubers of *Dioscorea bulbifera*, determining some of its physicochemical properties, identifying and sequencing the gene encoding the storage protein and establishing the phylogenetic relatedness with the storage proteins from other Dioscorea species.

## Methods

### Materials

*Dioscorea bulbifera* aerial tubers were obtained from a farmland in Amichi, Nnewi South Local Government of Anambra State, Nigeria. The plant was identified in the IFE Herbarium of the Department of Botany, Obafemi Awolowo University, Ile-Ife, Nigeria, where the specimen copy was deposited and voucher number IFE-14754 was given.

All chemicals and reagents used were purchased from either Sigma Chemical Co. (St. Louis, MO, USA), Pharmacia Chemicals (Uppsala, Sweden) or Bio-Rad Lab (Hercules, CA, USA).

### Preparation of crude extracts

The crude extract of the aerial tubers of *Dioscorea bulbifera* was prepared at different pH and varying temperature, in order to ascertain conditions at which most of the proteins in the aerial tuber are solubilized.

*Dioscorea bulbifera* aerial tubers were peeled, sliced and homogenized with 4 volumes (w/v) of buffers at different pH: 0.5 M citrate/phosphate buffer (pH 4–6), 0.5 M Tris-HCl buffer (pH 7 and pH 8.3) and glycine-NaOH buffer (pH 9 and 10). The mixtures were stirred for 4 h and centrifuged at 13,500 rpm for 30 min at 4 °C. The supernatants collected were stored as crude extracts.

Also, approximately 100 g portions of yam slices were boiled in 1 L of water at 25, 30, 40, 50, 60, 70, 80, 90 and 100 °C for 10 min. The treated aerial tubers were drained, cooled, weighed and homogenized with 50 mM Tris-HCl (pH 8.3) at 1:4 (w/v). The mixture was stirred for 4 h and centrifuged at 13,500 rpm for 30 min at 4 °C. The supernatants were collected as crude extracts.

Protein content of extracts was determined by Lowry method using 1 mg/mL bovine serum albumin (BSA) as standard.

### Purification of protein

The crude extract obtained at pH 8.3 and 25 °C (which had the highest protein concentration) was used for further studies. Purification of the storage protein of *Dioscorea bulbifera* aerial tuber was carried out following the method of Hou et al. [18] with a little modification.

The crude extract of the aerial tuber of *Dioscorea bulbifera* was subjected to 70% ammonium sulphate precipitation, stirred and kept overnight at 4 °C. The mixture was centrifuged at 13,500 rpm for 30 min and the precipitate recovered. The precipitate was dissolved in 10 volumes of 50 mM Tris-HCl buffer, pH 8.3 and dialyzed exhaustively against distilled water.

#### Ion-exchange chromatography on DEAE Sephadex A-25

The dialyzed protein solution (7.5 mg/mL; 2.5 mL) was loaded on DEAE Sephadex (A-25) ion exchange column (1.5 × 20 cm) previously equilibrated with 50 mM Tris-HCl buffer, pH 8.3. Unadsorbed proteins were eluted with 50 mM Tris-HCl buffer, pH 8.3, and adsorbed proteins were eluted stepwise with 150 mM NaCl in 50 mM Tris-HCl buffer, pH 8.3, at a flow rate of 15 mL/h. Fractions of 5 mL each were collected, and elution was monitored at 280 nm. The adsorbed protein fractions, which correspond to the major storage protein of the aerial tuber of *D. bulbifera*, were pooled and concentrated.

#### Gel filtration on Sephadex G-75

Adsorbed protein sample (1.5 mg/mL; 5 mL) obtained from ion-exchange chromatography was further purified by gel filtration on Sephadex G-75 column (1.5 × 40 cm) previously equilibrated with 50 mM Tris-HCl buffer, pH 8.3. The column was eluted with 100 mM Tris-HCl buffer (pH 7.9) containing 100 mM NaCl at a flow rate of 27 mL/h. Fractions of 3.6 mL each were collected. The purified protein was collected, concentrated and stored at − 20 °C for further use. Protein concentration was determined after each purification step.

### Non-SDS polyacrylamide gel electrophoresis

The protein samples were subjected to polyacrylamide gel electrophoresis in the absence of sodium dodecyl sulphate (SDS) according to the modified method of Shiu et al. [19] to monitor the purity of the protein obtained after each purification step. Electrophoresis was performed on a 10% discontinuous gel system under non-denaturing conditions and stained with Coomassie Brilliant Blue.

### Determination of molecular weight

The native molecular weight of the protein was determined by gel filtration on a Bio gel P-200 column (1.5 × 63 cm) using the following protein markers: lysozyme (Mr 14,000), α-chymotrypsinogen A (Mr 25,000), egg ovalbumin (Mr 45,000) and bovine serum albumin (Mr 66,000). Each protein (5 mL) was applied on the column and run separately using 10 mM phosphate buffer pH 7.0 as eluant at a flow rate of 10 mL/h. Fractions of 5 mL were collected, and the elution was monitored at 280 nm. The void volume (*V*_o_) of the column was determined using Blue dextran (elution monitored at 620 nm).

The purified storage protein was subjected to SDS-polyacrylamide gel electrophoresis for subunit molecular weight determination following the modified method of Shiu et al. [19] using the following protein markers: ovalbumin (Mr 45,000), carbonic anhydrase (Mr 29,000), trypsinogen (Mr 24,000), trypsin inhibitor (Mr 20,000) and α-lactalbumin (Mr 14,200).

### Detection of protein-bound carbohydrate

The presence of covalently-bound carbohydrate in the storage protein was investigated by staining the gels with periodic acid-Schiff’s reagent (PAS) after electrophoresis, as described in the Pharmacia Manual of Laboratory Techniques, revised edition. The protein sample was subjected to electrophoresis under non-denaturing conditions using phosphate-buffered system. After electrophoresis, the gel was fixed in 7.5% acetic acid at room temperature for 1 h. The fixed gel was transferred into a beaker containing 0.2% aqueous periodic acid and kept at 4 °C for 45 min. Afterwards, the gel was removed and transferred into a beaker containing Schiff’s reagent, kept at 4 °C for 45 min. The gel was destained in 10% acetic acid. Glycoprotein band (if present) will stain purplish red.

### Amino acid composition of the protein

The storage protein was subjected to amino acid content analysis using methods described by Ekeanyanwu [20]. The sample was hydrolysed, evaporated in a rotary evaporator and loaded into the Technicon Sequential Multi-Sample Amino Acid Analyzer (TSM).

### Enzymatic activities of storage protein of *Dioscorea bulbifera*

#### Determination of carbonic anhydrase activity

Carbonic anhydrase activity of the protein was measured by hydrolysis of 4-nitrophenyl acetate resulting in an increase of absorbance at 348 nm [21]. The activity of the tuber storage protein was compared with that of carbonic anhydrase from bovine erythrocytes. The reaction mixture contained 0.3 mL of freshly prepared 3 mM 4-nitrophenyl acetate in aqueous 3% acetone and 0.7 mL of 15 mM Tris sulphate buffer, pH 7.6. Exactly 10 μL purified protein solution (1 mg/mL) was added, and the catalyzed reaction was monitored by measuring the increase in absorbance at 348 nm for 5 min.

#### Determination of dehydroascorbate reductase activity

Dehydroascorbate (DHA) reductase activity of the protein was carried out according to the method of Hou et al. [18]. In this reaction, 10 mg of DHA was dissolved in 5 mL of 100 mM phosphate buffer of different pH values (pH 6.0, 6.5 and 7.0). The reaction was carried out at 30 °C; 100 μL purified protein solution (1 mg/mL) was added to 0.9 mL DHA solution with or without 4 mM glutathione. Increase in absorbance at 265 nm was recorded for 5 min. Non-enzymatic reduction of DHA in phosphate buffer was measured in a separate cuvette.

#### Determination of monodehydroascorbate reductase activity

Monodehydroascorbate (MDA) reductase activity of the protein was assayed according to the method described by Hou et al. [18] by monitoring the decrease in absorbance at 340 nm due to NADH oxidation. MDA free radicals were generated by ascorbate oxidase in the assay system. The reaction mixture contained 50 mM phosphate buffer (pH 6.0, 6.5 and 7.0); 0.33 mM NADH; 3 mM ascorbate, ascorbate oxidase (0.9 U); and 200 μL purified protein solution (200 μg protein) in a final volume of 1 mL. Distilled water was used to replace protein solution in blank solutions. One unit of MDA reductase is defined as the amount of protein required to oxidize 1 μmol of NADH per min.

#### Determination of trypsin inhibitory activity

Trypsin inhibitory activity of the protein was determined according to the method of Xue et al. [22] by monitoring the inhibition of trypsin-catalyzed hydrolysis of *N*-benzoyl-L-arginine-4-nitroanilide (substrate) in 0.1 M Tris-HCl buffer (pH 8.2). Different concentrations of the protein were pre-incubated with 20 μM trypsin at room temperature for 15 min. The substrate (100 μg/mL) was added to give a final volume of 1 mL for an additional 20 min. The absorbance at 405 nm was measured. The inhibitory activity is calculated as the percentage decrease in substrate hydrolysis rate, which is directly proportional to increase in absorbance at 405 nm. The result was expressed as micrograms of trypsin inhibited.

### Molecular characterization of the storage protein of *Dioscorea bulbifera*

#### Genomic DNA extraction

The aerial tuber was peeled, cut into bits and ground into fine powder with a mortar and pestle under liquid nitrogen. Genomic DNA was extracted using QIAGEN DNeasy Plant Mini Kit. DNA concentrations were determined with a Nanodrop spectrophotometer (Beckman Coulter) and adjusted to 25 ng/μL for PCR amplification.

#### Primer design for polymerase chain reaction

Sequences of some Dioscorin genes from various Dioscorea sp. were obtained from NCBI nucleotide database (http://www.ncbi.nlm.nih.gov/nuccore). These sequences were inserted into the input window of the web-based polyacrylamide chain reaction (PCR) primer designing program, Primer3 (https://primer3plus.com/cgi-bin/dev/primer3plus.cgi). The primer minimum and maximum sizes were set to 100 and 900 nucleotides, respectively. The DNA was subjected to PCR amplifications using the designed Dioscorin-specific primers (5′-CTCCTCTCCTCCCTCCTCTT-3′ (forward primer) and 5′-GGGGGTACAATGGAGAAGT G-3′ (reverse primer)). The amplification was conducted in a final reaction volume of 25 μL containing 5 μL of DNA sample, 2.0 μL MgCl_2_, 0.2 μL *Taq* polymerase, 2.5 μL 10 X reaction buffer, 1 μL dNTPs, 1 μL each of forward and reverse primers, 2.0 μL Tween 20 and sterile deionized water in a 96-well microtiter plate and carried out in a GeneAmp PCR System 9700 (Applied Biosystems). The PCR cycles were made up of initial denaturation of DNA template at 94 °C for 3 min, followed by 36 cycles of denaturation at 94 °C for 1 min, annealing at 60 °C for 1 min and extension at 72 °C for 2 min. The final extension step was at 72 °C for 7 min.

#### Electrophoresis of PCR products

The PCR products obtained were detected by agarose gel electrophoresis. An aliquot (3 μL) of 5 × loading dye (0.25% bromophenol blue, 0.25% xylene cyanol FF and 13% Ficoll in water) was added to the 10 μL of the PCR product, and 6 μL of the mixture was loaded onto a 1.5% agarose gel pre-stained with ethidium bromide. TBE (0.5X) was used as running buffer, and DNA ladder (markers) was loaded for fragment sizing. Electrophoresis was conducted at 1500 V for 3 h, and the gels were viewed under ultraviolet rays.

#### Gel extraction, DNA sequencing

The resulting DNA fragments generated from amplifications were purified by excising bands from the agarose gel after electrophoresis. The DNA was recovered using QIAquick gel extraction kit (Qiagen). The nucleotide sequences of the purified dioscorin genes were obtained with a genetic analyser. The sequencing amplifications were performed in a 20-μL reaction mixture consisting of 400 ng of DNA to be sequenced, 10 pmole of dioscorin-specific primers 5′-CTCCTCTCCTCCCTCCTCTT-3′ (forward primer) and 10 pmole of 5′- GGGGGTACAATGGAGAAGTG-3′ (reverse primer) and 4 μL of Reaction Dye Terminator Premix (Qiagen) with standard sequencing conditions. Amplification was performed in a thermowell microtitre plate (Costa Corporation) using Perkin Elmer programmable Thermal Controller model 9600. The cycling program was 36 cycles of 94 °C for 1 min for denaturation, 60 °C for 1 min for annealing of primers and 72 °C for 2 min for extension. Amplification products were stored at 4 °C before use. One microlitre of 125 mM EDTA, 1 μL 3 M sodium acetate (pH 4.8), 25 μL 100% ethanol (− 20 °C) and 50 μL 70% ethanol (− 20 °C) were added to the amplification products, mixed and centrifuged for 10 min at 10,000 rpm at 4 °C. DNA pellet was dried at room temperature and re-suspended in 5 μL sterile deionized distilled water. One microlitre of the re-suspended DNA was added to 9 μL Hi formamide, mixed and denatured for 3 min at 94 °C. It was placed inside ABI PRISM 3130 X1 genetic analyser, which carried out the automated sequencing analysis using a standard sequencing module with Performance Optimized Polymer and 50-cm array.

#### Sequence analysis

The nucleotide sequences of the purified dioscorin genes were subsequently translated to protein sequence using bioinformatic resource tool from CLC Genomics Workbench software (CLC Bio Denmark). The nucleotide and translated protein sequences were further subjected to computer-based homology search with NCBI BLAST program. Phylogenetic analysis was carried out to compare the relationship of the major storage protein of *D. bulbifera* with the storage proteins of other Dioscorea spp.

## Results

### Crude extracts

**Fig. 1:**
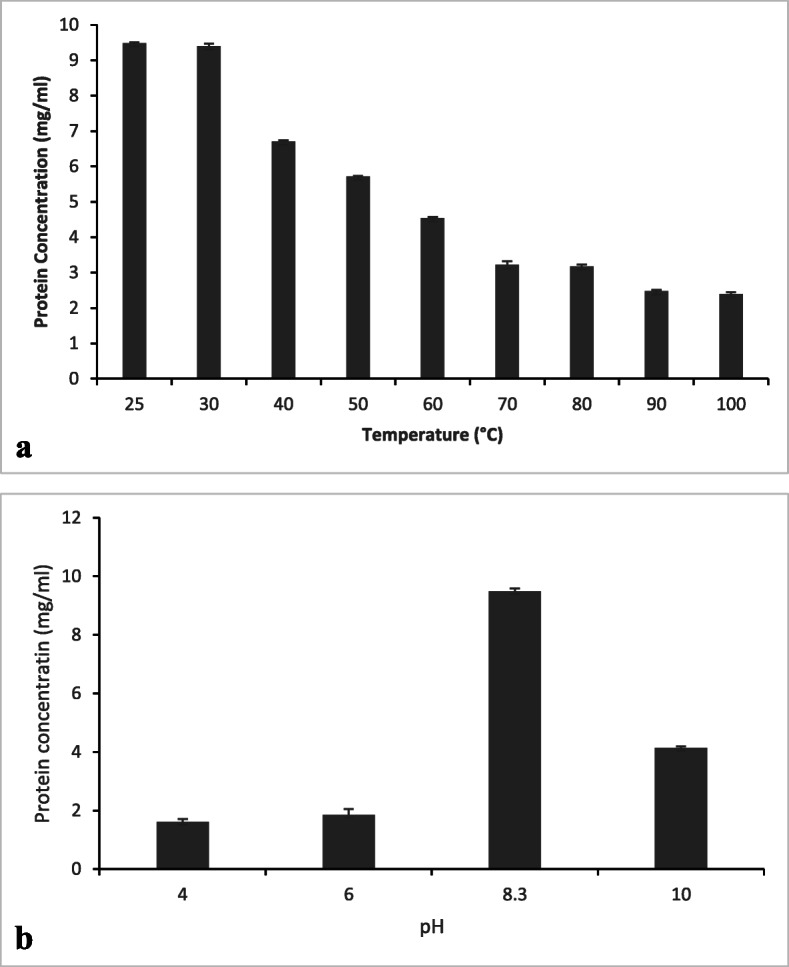
Protein concentration of the crude extracts of *Dioscorea bulbifera***a** at different temperature and **b** at different pH

The protein concentration of crude extracts of the aerial tuber of *Dioscorea bulbifera* extracted at different temperature and varying pH is as shown in Fig. 1. The result showed that maximum protein concentration was obtained at pH 8.3 and at room temperature, 25 °C.

### Purification of storage protein

**Fig. 2:**
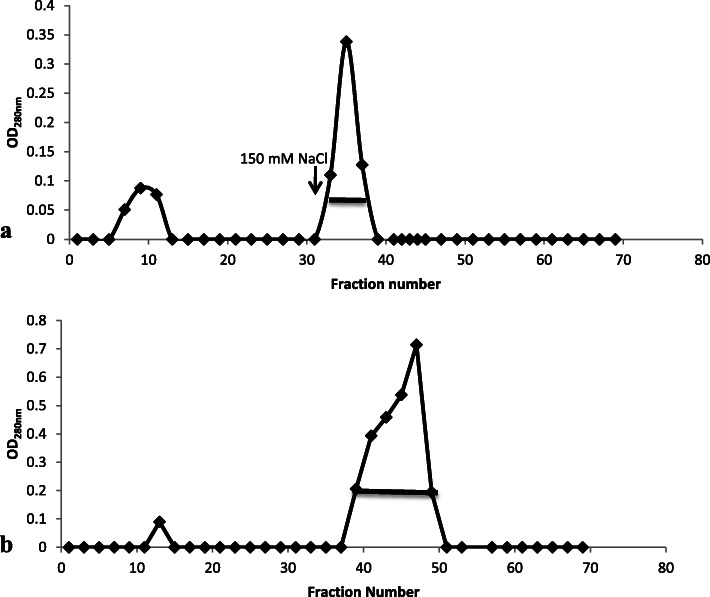
Column chromatography profile of *Dioscorea bulbifera.***a** Ion-exchange chromatography of dialyzed 70% ammonium sulphate precipitate of the extract of the aerial tuber of *Dioscorea bulbifera* on DEAE Sephadex A-25 column (elution buffer: 0.05 M Tris-HCl buffer pH 8.3. Adsorbed protein was eluted with 0.05 M Tris-HCl buffer pH 8.3, containing 150 mM NaCl). Column size (1.5 × 20) cm; flow rate 15 ml/h; fraction size 5 ml. **b** Gel filtration of adsorbed peak from ion-exchange on Sephadex G-75 column (elution buffer: 100 mM Tris-HCl pH 7.9 with 100 mM NaCl). Column size (1.5 × 40) cm; flow rate 27 ml/h; fraction size 3.6 ml

The crude extract of the aerial tuber of *Dioscorea bulbifera* (379.60 mg) when subjected to 70% ammonium sulphate precipitation gave 130.80 mg protein which corresponds to 34.46% yield. The elution profile of the ion-exchange chromatography on DEAE-Sepahdex A-25 of the dialyzed protein sample is as presented in Fig. 2a. Two protein peaks were obtained from the DEAE-Sephadex A-25 column, one unadsorbed peak and the adsorbed protein peak which was eluted with 150 mM NaCl. The adsorbed peak which was pooled and further purified by gel filtration on Sephadex G-75 column is as shown in Fig. 2b. At the end of purification, the amount of protein recovered was 13.20 mg, corresponding to 3.48% of the starting material.

### Molecular weight of *D. bulbifera* storage protein

**Fig. 3:**
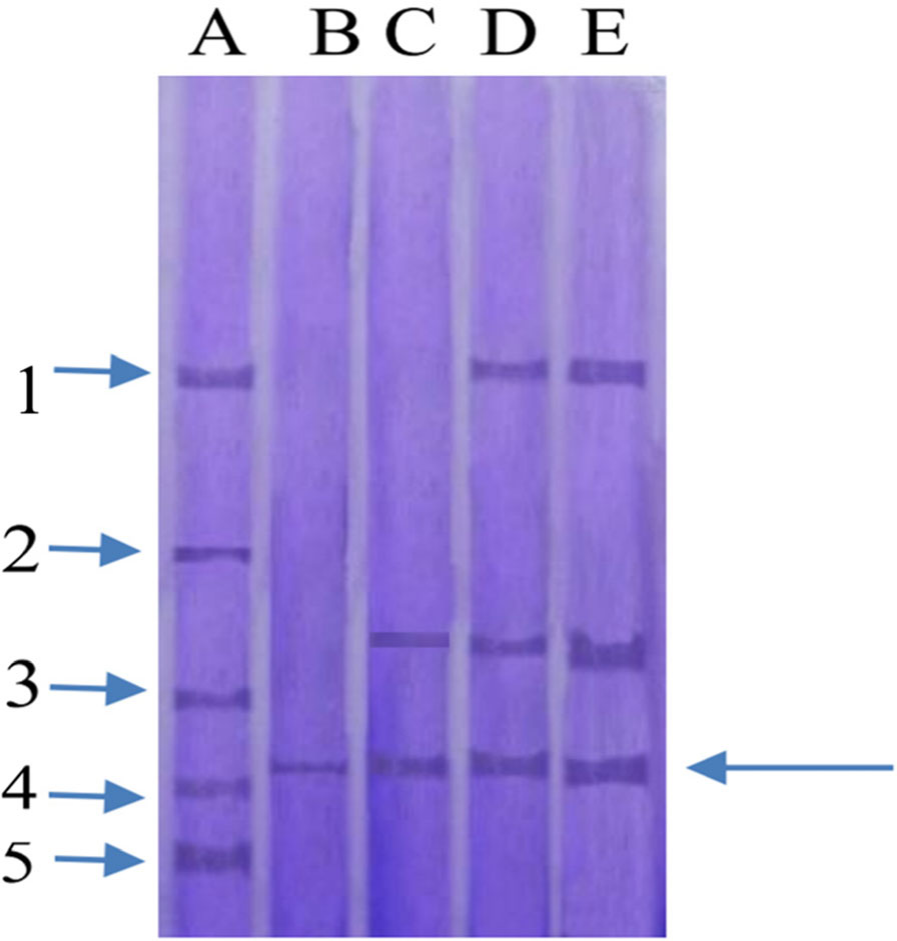
Electrogram of SDS-polyacrylamide gel electrophoresis of the storage protein of the aerial tuber of *D. bulbifera*. PAGE was carried out in the presence of SDS using the discontinuous Tris-glycine buffer system. The separating gel was 10%, and stacking gel was 3.5% acrylamide. Other conditions were as described in text. Lane A: standard proteins; B: gel filtration pooled fractions; C: ion exchange pooled fractions; D: ammonium sulphate precipitate; E: crude extract

The molecular weight of the native storage protein of the aerial tuber of *D. bulbifera* as determined by gel filtration on Bio-gel P-100 was 22,000 Da. The subunit molecular weight, which was determined by SDS-PAGE under denaturing conditions, was estimated to be 21,095 Da (Fig. 3).

### Detection of protein-bound carbohydrate

The storage protein of the aerial tuber did not stain purplish-red with Schiff’s reagent suggesting that it has no covalently linked carbohydrate molecule and thus is not a glycoprotein.

### Amino acid composition

The amino acid composition of the storage protein of the aerial tuber of *D. bulbifera* is presented in Table 1. The amino acid composition is characterized by an abundance of neutral and charged polar amino acids, especially tyrosine, arginine, glutamate and cysteine, which constituted about 58% of the total concentration amino acids of the protein (g/100 g protein). Among the non-polar amino acids, proline and phenylalanine were present in relatively high concentration. Of the sulphur-containing amino acids, concentration of cysteine was higher when compared with methionine. Tryptophan, which was probably destroyed during acid hydrolysis of the protein, was not detected.

**Table 1:** Amino acid composition of the storage protein of *Dioscorea bulbifera*

Amino acid	Concentration (g/100 g protein)
Lysine	3.50
Histidine	4.43
Arginine	11.87
Aspartic acid	4.41
Threonine	3.97
Serine	4.10
Glutamic acid	9.92
Proline	16.20
Glycine	4.39
Alanine	5.79
Cysteine	9.66
Valine	4.25
Methionine	3.75
Isoleucine	4.56
Leucine	4.09
Tyrosine	23.11
Phenylalanine	12.29
Tryptophan	ND

### Carbonic anhydrase activity

The protein had low carbonic anhydrase activity (0.202 units/mg) as compared with standard carbonic anhydrase from bovine erythrocytes.

### Dehydroascorbate reductase activity

**Fig. 4:**
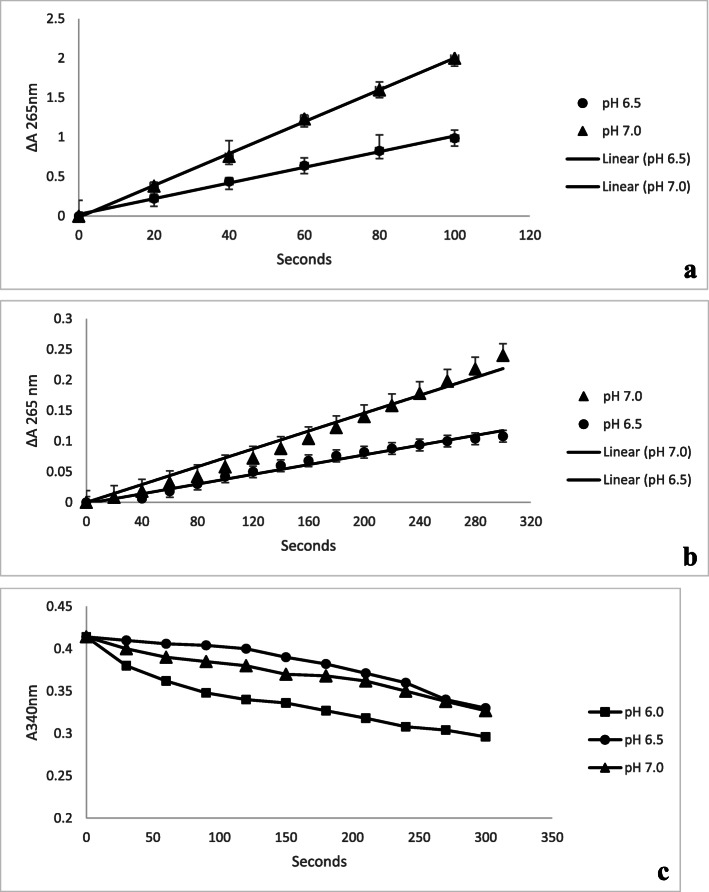
Dehydroascorbate reductase activity of storage protein of the aerial tuber of *D. bulbifera* at pHs 6.5 and 7 with (**a**) or without (**b**) 4 mM glutathione and (**c**) monodehydroascorbate reductase activity of the storage protein of the aerial tuber of *D. bulbifera* at pHs 6, 6.5 and 7

The storage protein of the aerial tuber of *D. bulbifera* exhibited dehydroascorbate reductase activity. The protein was able to regenerate ascorbate from dehydroascorbate in the presence and absence of glutathione as shown in Fig. 4a, b. In the presence of glutathione, the specific activities of dehydroascorbate reductase for the protein were 4.14 and 6.01 μmol ascorbic acid produced/min/mg protein at pH 6.5 and pH 7.0, respectively. In the absence of glutathione, the specific activities were 2.07 and 2.76 μmol ascorbic acid produced/min/mg protein at pH 6.5 and pH 7.0, respectively. No activity was observed at pH 6.0.

### Monodehydroascorbate reductase activity

The storage protein from the aerial tuber of *D. bulbifera* showed monodehydroascorbate reductase activity. The protein reduced monodehydroascorbate to ascorbate coupled with NADH oxidation. At pH 6.0, the activity was 0.0017 units/mg which implies that the amount of protein required to oxidize 1 μmol of NADH per min at pH 6.0 was 0.0017 units/mg. At pH 6.5 and pH 7.0, the activity was 0.00038 units/mg and 0.00051 units/mg respectively. Monodehydroascorbate reductase activity was higher at pH 6.0 than at other pH as shown in Fig. 4c.

### Trypsin inhibitory activity

**Fig. 5:**
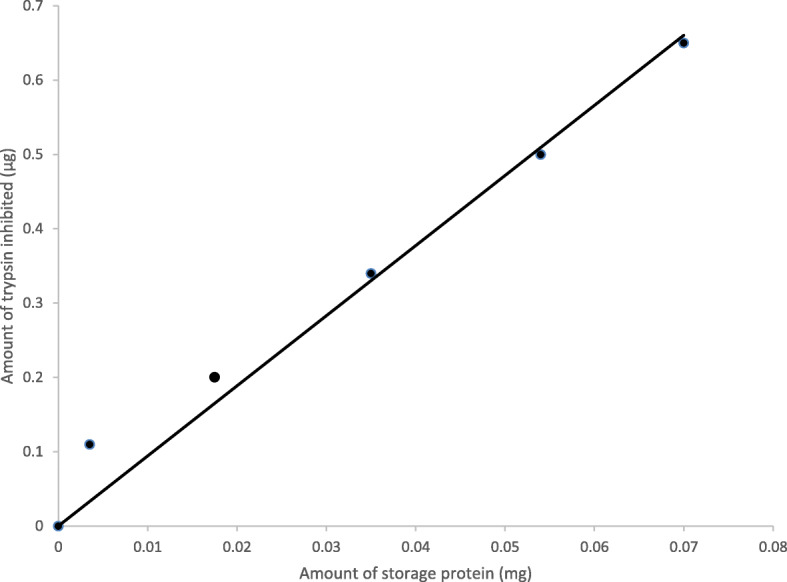
Trypsin inhibitory activity of the major storage protein of the aerial tuber of *D. bulbifera*

Different amounts of the protein were used to determine trypsin inhibitory activity, and the activity was expressed as micrograms of trypsin inhibited as shown in Fig. 5. A positive correlation (*r*^2^ = 0.9752) was found between trypsin inhibitory activity and amounts of storage protein from the aerial tuber of *D. bulbifera*. The storage protein of the aerial tuber of *D. bulbifera* exhibited low trypsin inhibitory activity with an average of 0.94 μg trypsin inhibited per 100 μg of the protein.

### Presence of dioscorin genes

**Fig. 6:**
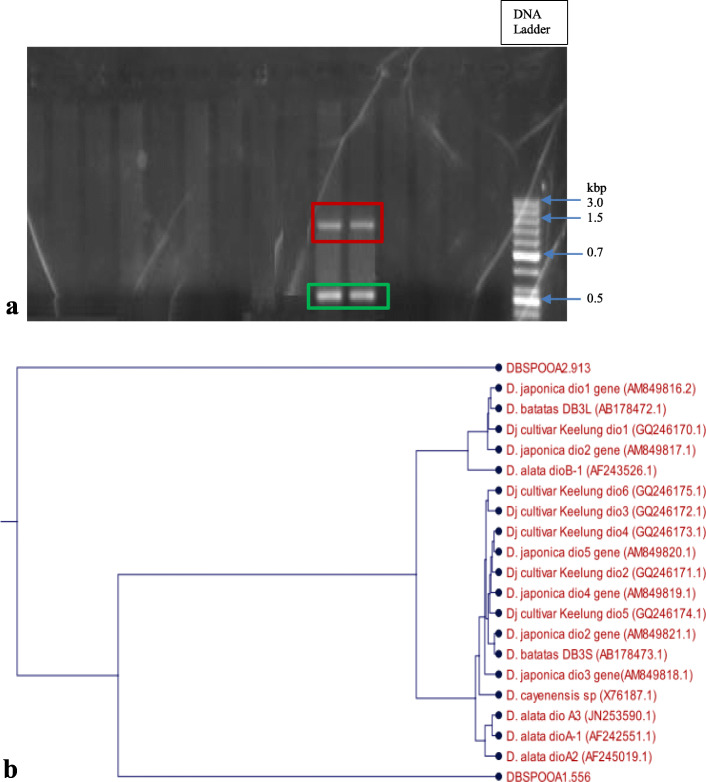
**a** PCR amplification of *D. bulbifera* Dioscorin genes (DBSPOOA) with Dioscorin specific primer set (5′-CTCCTCTCCTCCCTCCTCTT-3′ and 5′-GGGGGTACAATGGAGAAGTG-3′). PCR products were resolved on agarose gels and stained with ethidium bromide. The last lane shows DNA ladder containing DNA fragments of defined length for sizing the bands in the experimental PCRs. The bands outlined in green and red represent DBSPOOA1 and DBSPOOA2, respectively. **b** Phylogenetic relationship between DBSPOOA genes and other Dioscorin genes

Screening of the primer sets designed for the study with the genomic DNA samples revealed some of the specific primers were able to detect the dioscorin gene in the genomic DNA sample of *Dioscorea bulbifera*. Target sequences are readily obtained by polymerase chain reaction if the flanking sequences of the target sequences are known. The presence of dioscorin gene was thus established in the genomic DNA extracted from the aerial tuber of *Dioscorea bulbifera*. Sequence analysis of dioscorin gene DNA marker produced two DNA fragments and nucleotide sequence sizes which were DBSPOOA1-556 bp and DBSPOOA2-913 bp, respectively (Fig. 6a).

### Homologous similarities of the genes and sequence alignments

BLAST homology search using nucleotide sequence of DBSPOOA1-556 gave significant alignments of 100% nucleotide identity with Dioscorin B from *Dioscorea alata* (dioB-1), 96% nucleotide identity with *Dioscorea oppositifolia* microsatellite Dios23 sequence and 88% nucleotide identity with *Arabidopsis thaliana* chromosome 3. DBSPOOA2-913 gave significant alignments of 91% nucleotide alignment with *D. alata* voucher GZY109 ribulose-1,5-bisphosphate carboxylase large subunit (rbcL) gene and 81% nucleotide identity with *D. japonica* voucher Hsu 231 ribulose-1,5-bisphosphate carboxylase large subunit (rbcL) gene (Table 2).

**Table 2:** Dioscorin gene DNA marker (DBSPOOA1-556) nucleotide sequence and deduced amino acid sequence alignments and homologous details

DBSPOOA1 gene	
Nucleotide sequence (556 bp)	CCTCTCTCTCACTCAACTTTTGCCGCATCCCCCCACCACTCCCCTCCCAGCACTCCCTCTCCTCTCCTTGCCCCCCTTCTACCCTCCTGCCCCCCCGTTCCAAAACTTCTTCTATTCCCCATCTGTTTTTACCAAAGATGATGATCCTTAACTTCTTCTTCCTCCACTTCTCCATTGTACCCCCTTAATTTTACGGCCAGTGGTTCACCAAAGGAAGAAAATTCTTATTGGTCGCCGCAAATCCGACGAGCTTGTTGAGCGTCATAGCCAATCTTGCCGGAGCAAGAGCTGCGGCAAGAATGGGATGGAAAGAGAGTGCGGTTTGCATGTTTTCACTAGCCATGACTCATTACCTCGTGTTATTCGTAACCTTGTATCAGCGTCTACAAGGCAGCAACAGCCTTCCGGCGATGCTCCGTCCGGCTTTCTTCCTCTTCTTCGCCGCACCGAGCATGGCCAGTTTCACCTGGGTGTCAATTTCCGGCGAATTCGACATCTCATGCAAAATGCTTTTCTTCCTTTCTCTTTTCCTCTTCACTTCTCCATTGTACCCCCT
Translated amino acids sequence (184 amino acids)	SLSLNFCRIPPPLPSQHSLSSPCPPSTLLPPRSKTSSIPHLFLPKMMILNFFFLHFSIVPPFYGQWFTKGRKFLLVAANPTSLLSVIANLAGARAAARMGWKESAVCMFSLAMTHYLVLFVTLYQRLQGSNSLPAMLRPAFFLFFAAPSMASFTWVSISGEFDISCKMLFFLSLFLFTSPLYP
Nucleotide sequence alignments	1. *Dioscorea alata* Dioscorin B (dioB-1) mRNA, complete cds; 100% identity; Accession (AF243526.1) 2. *Dioscorea oppositifolia* microsatellite Dios23 sequence; 96% identity; Accession (JQ955632.1) 3. *Dioscorea cirrhosa* isolate PS5034MT03 ribulose-1,5-bisphosphate carboxylase/oxygenase (rbcL) gene, partial cds; chloroplast; 95% identity; Accession (HQ637837.1) 4. *Dioscorea alata* voucher GZY110 ribulose-1,5-bisphosphate carboxylase/oxygenase large subunit (rbcL) gene, partial cds; chloroplast; 95% identity; Accession (JX139768.1) 5. *Ipomoea batatas* voucher GZY116 ribulose-1,5-bisphosphate carboxylase/oxygenase large subunit (rbcL) gene, partial cds; chloroplast; 95% identity; Accession (JX139773.1) 6. *Arabidopsis thaliana* chromosome 3, complete sequence; 88% identity; Accession (CP002686.1)
Translated amino acids sequence alignments	1. Dioscorin B [*Dioscorea alata*]; 75% identity; Accession (AAF44711.1) 2. PREDICTED: S-type anion channel SLAH1-like [*Solanum tuberosum*]; 73 % identity; Accession (XP_006342228.1) 3. Hypothetical protein OsJ_01095 [*Oryza sativa Japonica* Group]; 71% identity; Accession (EAZ11241.1) 4. S-type anion channel SLAH1 [*Arabidopsis thaliana*]; 68% identity; Accession (NP_176418.2) 5. Storage protein [*Dioscorea cayenensis*]; 60% identity; Accession (CAA53781.1)

BLAST homology search using translated amino acid sequences from the nucleotide sequences of DBSPOOA1-556 and DBSPOOA2-913 also produced sequence homology with known protein sequences from *Dioscorea* spp. DBSPOOA1-556 conceptual amino acid sequence has 75% amino acid sequence identity with Dioscorin B from *D. alata*, 73% identity with the predicted S-type anion channel SLAH1-like from *Solanum tuberosum* (potato), 71% identity with the hypothetical protein OsJ_01095 of *Oryza sativa Japonica* (rice) group, 68% identity with S-type anion channel SLAH1 of *A. thaliana* and 60% amino acid sequence identity with the storage protein of *Dioscorea cayenensis*. The minimum molecular weight calculated from translated sequence of DBSPOOA1-556 using Protparam online server (https://web.expasy.org/protparam/) is 20,456.38 Da, which is similar to what was obtained for the subunit molecular weight of the storage protein by SDS-PAGE (21,095 Da). DBSPOOA2-913 conceptual amino acid sequence also showed homology with other known proteins, such as ribulose-1,5-bisphoshate carboxylase/oxygenase large subunit of *D. bulbifera*, putative carbonic anhydrase of *Neosartorya fischeri* and putative Dioscorin from *O. sativa Japonica* group (Table 3).

**Table 3:** Dioscorin gene DNA marker (DBSPOOA2-913) nucleotide sequence and deduced amino acid sequence alignments and homologous details

DBSPOOA2 gene	
Nucleotide sequence (913 bp)	ACCCTCATGGGTGTCGGTGAGGAGAAGGTGACCCGGCAGCGGAGTTTGTTCGCAGGAGATAGAGGAGTCCAGGGAGACCTACGGTGGGCTGTTTATGAATGTCTACGTGGGGGACTTGATTTTACCAAAGATGATGACGCTGATGAAGGGGGGGAAAAGCACGGGAGGGTGAGTCATGGCTGGTGAAAACTTGCTAACCGCACTCTCTTTCCATCCCATTCTGGCCGCAGCTCTTGCTCCGGCAAGATTGGCTATGACGCTCAACAAGCTCGGCGGATTTGCGGCGACCAATAAGAATTTTCTTCCTTTGGGGAACCACTGGTCGTAGATTTTTACGTCGAGGACAATTACCGGGAGCGAAAAGAAGAGACACACAAGAATGAACGGAGGAGATTGAGGATCAAGAAAAGGAGTTGATTGGAGAAGGAGCAAGAAAGAATTCCAAGGTGCGAATAGATAGTTCATGCCGATATAGTCAGACAACTCGGCGCGAACATGGTGGAACTGGCGGAGGCACCGGAGGAGGAAGAGGAGGGAGGAGAGGAGAACTATTTCTCTGCTTTACCTCCAATTATCATCTAATGTGATTCCCTCCTTCCATAAAATCCATCTTATCTTTATTAAAATGGGTGCTCTTCTACCTGCTGCTAATATTCCATAAACCACTTGATAACCTGAATTTATTTATCTTTATCCTACTGTACAAGGCATCTTAGAAAAAGCGTTGTTTCCTTCTTTCTACCACATCCAACTTGGATTGTTATTCCCTTCCTTTGCAAAATTTATATAAGATTTTTTTTTCCTCTCGAATAACCCTGTGACCCCTTTAGCTGGGTGAACTTTTCACCATTCGGACATGCTCTCCTGTACCATATTTTTCTTTGTGCCTGCTTCCTATTTACCCCTCCCACAA
Translated amino acid sequence (304 amino acids)	TLMGVGEEKVTRQRSLFAGDRGVQGDLRWAVYECLRGGLDFTKDDDADEGGEKHGRVSHGWKLANRTLFPSHSGRSSCSGKIGYDAQQARRICGDQEFSSFGEPLVVDFYVEDNYRERKEETHKNERRRLRIKKRSLEKEQERIPRCEIVHADIVRQLGANMVELAEAPEEEEEGGEENYFSALPPIIICDSLLPNPSYLYNGCSSTCCYSINHLITIYLSLSYCTRHLRKSVVSFFLPHPTWIVIPFLCKIYIRFFFPLEPCDPFSWVNFSPFGHALLYHIFLCACFLFTPPT
Nucleotide sequence alignments	1. *Dioscorea japonica* voucher Hsu 231 ribulose-1,5-bisphosphate carboxylase/oxygenase large subunit (rbcL) gene, partial cds; chloroplast; 89% identity; Accession (JQ733767.1) 2. *Dioscorea alata* voucher GZY109 ribulose-1,5-bisphosphate carboxylase/oxygenase large subunit (rbcL) gene, partial cds; chloroplast; 91% identity; Accession (JX139767.1) 3. *Dioscorea nitens* voucher YSL 2628 ribulose-1,5-bisphosphate carboxylase/oxygenase large subunit (rbcL) gene, partial cds; chloroplast; 89% identity; Accession (JQ733810.1)
Translated amino acid sequences alignments	1. Putative Dioscorin [*Oryza sativa Japonica* Group]; 41% identity; Accession (BAC99799.1) 2. Ribulose-1,5-bisphosphate carboxylase/oxygenase large subunit [*Dioscorea esculenta*]; 94% identity; Accession (AFC89185.1) 3. *Carbonic anhydrase*, putative [*Neosartorya fischeri* NRRL 181]; 45% identity; Accession (XP_001267068.1) 4. Ribulose-1,5-bisphosphate carboxylase/oxygenase large subunit [*Trebouxia simplex*]; 94% identity; Accession (AIJ50559.1)

Relationship among the storage protein gene from the aerial tuber of *D. bulbifera* (DBSPOOA1-556 and DBSPOOA2-913) obtained in this study and the storage protein genes from other *Dioscorea* spp. was revealed by CLCBio homology nucleotide sequence alignment unweighted pair-group method arithmetic (UPGMA) analysis. The analysis revealed that DBSPOOA1-556 and DBSPOOA2-913 are distinctly different. However, DBSPOOA1-556 formed a cluster with other Dioscorin genes from different *Dioscorea* spp. from Asian countries, but DBSPOOA2-913 was distinctly different from all known Dioscorin genes as shown in Fig. 6b.

## Discussion

The crude extracts of fresh aerial tuber of *D. bulbifera* prepared at 25 °C and at pH 8.3 showed optimum protein concentration, which is similar to the results obtained from previous reports [23, 24] on the effect of heating temperature and pH on the major storage protein of various yam species. There was reduction in protein concentration of *Dioscorea alata* L. var. *purpurea* at increasing temperature, and at temperature above 90 °C, there was complete denaturation of the protein. Protein concentrations of *D. alata* L. var. Tainung No. 2 and *D. japonica* Thunb. Var. *pseudojaponica* showed similar trend with *D. alata* L. var. *purpurea* with increasing heating temperatures except that the storage proteins were not extractable at temperatures above 80 °C. Protein concentrations of the yam storage proteins were not changed after heating at temperatures between 30 and 40 °C [23]. Protein concentration of *D. bulbifera* storage protein was highest at pH 8.3 which compares reasonably with what was obtained for the major storage proteins of other yam tubers [18, 24–26]. At acidic medium, low protein concentrations were observed for *D. alata* L. var. *purpurea*, *D. alata* L. var. Tainung No. 2 and *D. japonica* Thunb. Var. *pseudojaponica* [24].

The major storage protein of the aerial tuber of *D. bulbifera* obtained was about 87% of the total protein of the aerial tuber which is similar to the percentage concentration of the major storage proteins from other yam tubers [27]. DB2, the major storage protein of *Dioscorea batatas*, accounted for 50% of the total protein of the tuber [21]. The methods of purification of the storage proteins follow similar trends of ammonium sulphate precipitation followed by ion-exchange chromatography and hydrophobic or gel filtration chromatography, or a combination of any two of these steps [21, 23]. However, some researchers purified the major storage proteins from the yam tubers using a one-step purification protocol either by ion exchange (most especially on DE-52 column) or gel filtration on Sephadex G-75 [28, 29].

The native molecular weight of *D. bulbifera* major storage protein was estimated to be 22,000 Da while the subunit molecular weight was 21,000 Da, suggestive of a monomeric structure for the protein. This result is in contrast with those obtained for other underground yam tuber storage proteins. Dioscorins purified from other yam tubers showed a number of isoforms of about 31,000 and 32,000 Da [11, 30]. The storage protein isolated from the tuber mucilage of *D. batatas* had molecular weight above 250,000 Da while that from *D. cayenensis* was 31,000 Da [29]. The dioscorins isolated from *D. batatas* showed two bands (28,000 and 82,000 Da) on non-reducing SDS-PAGE and only one band (32,000 Da) under reducing condition [25]. On the other hand, *D. alata* was reported to have four subunits with molecular weight of 32,000 Da [31], while the storage protein of *Dioscorea opposita* was a monomeric protein with molecular weight of 32,000 Da [32]. Wang et al. [33] also purified a 32,000-Da storage protein from *D. purpurea.* Different yam cultivars have therefore been reported to behave differently in protein composition and structure [34].

*D. bulbifera* storage protein is not glycosylated as shown by periodic acid Schiff’s reagent (PAS) staining technique. Storage proteins from *D. batatas* and *Dioscorea rotundata* were also reported not to be glycosylated with PAS staining method [21]. On the contrary, the yam storage proteins from *D. batatas*, *D. alata* cv. Tainong No. 1 [27] and *D. japonica* [22] were reported to be glycosylated using conA-peroxidase staining method. The protein from *D. opposita* was also shown to be glycosylated with PAS staining [32].

Amino acid composition analysis of *D. bulbifera* storage protein revealed that it is characterized by high content of tyrosine, proline, phenylalanine, cysteine, glutamic acid and arginine. The high content of cysteine residues showed some similarity with the dioscorins from *D. batatas* and *D. japonica* [22, 25, 34] with high half-cystine content. In contrast, dioscorins from four cultivars of *D. alata* (Tainung No. 1, Tainung No. 2, Dasan and Chanhon) had only trace amounts of cysteine [35]. Cysteine, a sulphur-containing amino acid, even though non-essential is required in the diet to meet the body’s requirement. Sulphur is an important element necessary for normal growth and metabolism. Cysteine has been implicated in anti-ageing, promoting healthy hair and skin and also boosts the immune system. Cysteine residues are also very important especially in crosslinking proteins, increasing the rigidity of proteins and also conferring proteolytic resistance. The storage protein of *D. bulbifera* contains high amounts of essential amino acids, approximately 40.9% of the total concentration of amino acids (g/100 g protein), especially phenylalanine and arginine.

The 22 kDa storage protein of the aerial tuber of *D. bulbifera* exhibited carbonic anhydrase activity, albeit low. Carbonic anhydrases are zinc metalloenzymes that catalyze the simple interconversion of CO_2_ and HCO_3_^−^. They are pH regulatory and metabolic enzymes found in almost all organisms. In higher plants, carbonic anhydrases play a vital role in CO_2_ fixation during photosynthesis [36]. In mammals, they are involved in respiration [37]. Carbonic anhydrases are found in many tissue where they participate in many biological processes such as acid-base regulation, respiration, carbon dioxide and ion transport, bone resorption, ureagenesis, gluconeogenesis, lipogenesis and electrolyte secretion. Thus, they are important therapeutic targets for treatments of derangements such as edema, glaucoma, obesity, cancer and epilepsy [38]. Six genetically distinct carbonic anhydrases gene families have been identified (α-, β-, γ- δ-, z- and η-carbonic anhydrases) [39, 40]. Hou et al. [25] showed that the major storage protein of *D. batatas* had carbonic anhydrase activity, which could not be detected in another study by Gaidamashvili et al. [21]. The discrepancies in the two reports, albeit, in the same yam species could not be explained. Also, carbonic anhydrase activity was detected in the major yam storage proteins from different species of *Dioscorea*, *D. alata* (var. Tainong 1, var. Tainong 2, var. Zhongguochang) and *D. pseudojaponica* var. Keelung [27]. Xue et al. [41] also revealed that yam storage proteins, dioscorins, catalyse reactions assumed by carbonic anhydrases.

Ascorbic acid (vitamin C) is a plant secondary metabolite involved in a number of physiological processes. The main role of ascorbic acid is to neutralize free radicals and prevent against oxidative damage [42]. It also functions as a cell signalling modulator in cell division, growth regulation and senescence in plants [43, 44]. Because of the deleterious effects of reactive oxygen species (mostly as a result of salt imbalance), plants usually have well-developed enzymatic and non-enzymatic antioxidant defense system [45]. In plants, enzymes involved in the ascorbate-glutathione pathway (ascorbic acid-specific peroxidase, monodehydroascorbate reductase, dehydroascorbate reductase and glutathione reductase) assist in peroxides (formed as by-products of normal metabolism or as a result of environmental stresses) detoxification [46]. In its role as an antioxidant, ascorbic acid is univalently oxidized to monodehydroascorbate, an endogenous index of oxidative stress, which in turn rapidly dissociates to form ascorbic acid and dehydroascorbate in a reaction catalysed by mondehydroascorbate reductase [46]. Thus, monodehydroascorbate reductase and dehydroascorbate reductase are important in the regulating ascorbic acid level and its redox state during oxidative stress [47]. The major storage protein of the aerial tuber of *D. bulbifera* was shown to have both dehydroascorbate reductase and monodehydroascorbate reductase activities. The dehydroascorbate reductase activity was higher in the presence of gluthathione. Dehydroascorbate reductase activity was also detected without gluthatione but was lower when compared to the activity in the presence of gluthathione. The activity was also found to be pH dependent. At pH 6.0, there was no activity detected without gluthatione. This is similar to the report of the yam storage proteins, dioscorins, of *D. batatas* tuber which displayed both dehydroascorbate reductase and monodehydroascorbate reductase activities with and without gluthathione [18]. These activities might represent an important defense in the cytoplasm of yam cells in response to environmental oxidative stress [18].

Most storage proteins have been reported to play protective roles against environmental stresses, such as acting as protease inhibitors [48]. Protease inhibitors in plants are usually termed as anti-nutritional compounds because of their ability to inhibit digestive enzymes. However, their presence in plants is often as a result of an evolutionary adaptation which allows plants to survive under natural conditions [49]. In plants, protease inhibitors may be important in regulating and controlling endogenous proteinases, serving as storage proteins, and acting as protective agents against insect and microbial proteases. Protease inhibitors have also been classified under potential cancer-protective micro-components, by controlling misfunctioning of certain proteases in cancer progression [49]. The N-terminal amino acid sequences of storage proteins purified from yam bean (*Pachyrhizus erosus*), YGB1 and YGB2, showed high homology to cysteine protease, but both of them exhibited low protease activities using azocasein as substrates [50]. The storage protein from the aerial tuber of *D. bulbifera* had low trypsin inhibitory activity as compared with those from sweet potato roots [48]. However, just like the result obtained from this study, dioscorin from *D. batatas* showed only a weak trypsin inhibitory activity, with 1.9 μg of trypsin inhibited per 100 μg of the protein [25]. Large amounts of these storage proteins could provide a significant protective role in the aerial tuber even with this low trypsin inhibitory activity.

Two dioscorin gene DNA markers were amplified from the total genomic DNA of the aerial tuber of *D. bulbifera*, DBSPOOA1-556 and DBSPOOA2-913 using primers designed with NCBI primer BLAST tool and Primer 3. In a study done by Barman et al. [51], dioscorin gene was also amplified from RNA extracted from different species and cultivars of *Dioscorea* using forward and reverse primers designed with NCBI primer BLAST tool and Primer 3 software. The deduced amino acid sequences from *D. bulbifera* genes gave sequences of 184 and 304 amino acid residues respectively. The deduced amino acid sequences from DBSP00A1-556 was highly homologous to the amino acid sequences from the storage proteins of *D. alata* (dio-B) (75% identity) and *D. cayenensis* (60% identity). The deduced amino acid sequences for the two genes did not show any similarities with tuber storage protein genes of patatin and sporamin. Proteins of the same family normally perform the same biochemical function and may be related phylogenetically [52]. Amino acid sequence analyses have been found necessary for unambiguous evidence of structural relationship among proteins. Proteins with structural similarities tend to have evolutionary and functional similarities. In addition to having sequence similarities with the storage proteins from *D. cayenensis* and *D. alata* (dio-B), the translated amino acid sequence from DBSPOOA1-556 had homologous sequences with the predicted S-type anion channel SLAH1-like from *Solanum tuberosum*, and S-type anion channel SLAH1 from *Arabidopsis thaliana*. The predicted S-type anion channel from *Solanum tuberosum* is a hypothetical protein OsJ_01095 from *Oryza sativa Japonica* group and the S-type anion channel SLAH1 (SLAC1-homolog protein 1) from *Arabidopsis thaliana* is a slow and weak voltage-dependent S-type anion efflux channel which is involved in the maintenance of anion homeostatis. Also, the translated amino acid sequence from DBSPOOA2-924 was highly homologous with a putative dioscorin from *Oryza sativa* Japonica group and putative carbonic anhydrase from *Neosartorya fischeri* NRRL 181. Of the two genes of the storage protein from the aerial tuber of *D. bulbifera*, DBSPOOA1-556 formed a cluster with other dioscorin genes from different *Dioscorea* spp. from Asian countries, but DBSPOOA2-913 was distinctly different from all known dioscorin genes. This could be because it is an aerial tuber and not an underground tuber. It could also be because of the different geographical locations in which the yam species are cultivated. Several factors such as root-crop species, local climate and fertilization pattern have been reported to directly influence the composition of root crops [35]. The yam species used in this study was cultivated in Africa in contrast to various reported studies of other yam species, *D. alata*, *D. cayenensis*, and *D. japonica* which are cultivated in Asian countries.

## Conclusion

In conclusion, a storage protein was isolated from the aerial tuber of *Dioscorea bulbifera* for the first time. The storage protein has similar functional properties and structural homology with the storage proteins of other *Dioscorea* species. The storage protein is heat stable and exhibited carbonic anhydrase, dehydroascorbate reductase and trypsin inhibitory activities. It is also a good source of essential amino acids; thus, the protein may be suitable for development as functional food.

## Data Availability

The datasets used and/or analysed during the current study are available from the corresponding author on reasonable request.
